# Survey of Knowledge, Attitudes, and Levels of Confidence Regarding Age-Related Hyperkyphosis and Its Management among Thai Physiotherapists

**DOI:** 10.3390/healthcare12191998

**Published:** 2024-10-07

**Authors:** Roongtip Duangkaew, Sutima Suwankan, Sirinee Piyamasikul, Tharudee Namburee, Panisara Kesornbuakhao, Arisa Kishi, Josette Bettany-Saltikov

**Affiliations:** 1 Department of Physiotherapy, Faculty of Allied Health Sciences, Thammasat University, Pathumthani 12121, Thailand; sirinee.piy@dome.tu.ac.th (S.P.); tharudee.nam@dome.tu.ac.th (T.N.); panisara.kes@dome.tu.ac.th (P.K.); arisa.kis@dome.tu.ac.th (A.K.); 2 Department of Sport Science and Sport Development, Faculty of Allied Health Sciences, Thammasat University, Pathumthani 12121, Thailand; sutima.sstu@allied.tu.ac.th; 3 School of Health and Life Sciences, Teesside University, Middlesbrough TS1 3BX, UK; j.b.saltikov@tees.ac.uk

**Keywords:** age-related hyperkyphosis, elderly, physiotherapy, knowledge, attitudes, confidence

## Abstract

Background: Thorough knowledge of the management of age-related hyperkyphosis is crucial to physiotherapists’ effective handling of complex spinal deformities. Objective: This cross-sectional study investigated the knowledge, attitudes, and confidence of Thai physiotherapists regarding age-related hyperkyphosis. Methods: An online questionnaire with demographic, work-related, knowledge, attitude, and confidence questions was employed. The data analysis involved descriptive statistics, chi-square tests, and independent *t*-tests. Results: Complete responses were obtained from 314 physiotherapists. The correct responses amounted to 68.79% for the definition, 90.00% for causes, 14.97% for age of onset, 22.38% for prevalence, and 64.65% for the gold-standard diagnosis of hyperkyphosis. Most of the diagnostic methods involved visual examination. The respondents reported that hyperkyphosis disrupts respiratory function. The aim of treatment, according to 88.85%, was to increase spine mobility. Corrective posture exercises were the most common treatment strategy, but the range of treatments was diverse. Many cited undergraduate education as their primary evidence source. Respondents had conflicting attitudes towards “age-related hyperkyphosis is a normal aging process” but mostly positive attitudes towards the need for physiotherapy treatment. Approximately 22% reported fair confidence in treating hyperkyphosis. Conclusions: This study highlights the importance of raising awareness and enhancing knowledge, attitudes, and confidence among physiotherapists to improve care for older adults with hyperkyphosis.

## 1. Introduction

Age-related hyperkyphosis is the most common spinal deformity observed in older adults and affects up to 40% of older adults [1,2,3]. Thoracic hyperkyphosis is characterized by an excessive anterior curvature of the thoracic spine greater than 40° using the Cobb method (the current gold standard for quantifying thoracic kyphosis) [3]. The etiology of hyperkyphosis is multi-factorial, including degenerative disk disease, proprioceptive deficits, decreased spinal mobility, weak back muscles, low bone mineral density, vertebral fractures, and genetics [3]. The cause of thoracic hyperkyphosis also relates to the inability of the antigravity system to maintain a proper posture. This system includes postural tension, organization of opposed innervation, and proper coordination of postural and motor patterns [4]. An increased thoracic kyphosis causes an anterior displacement of the body’s center of mass closer to its stability limits, which affects balance, physical function, and activities of daily living [5,6]. Furthermore, excessive thoracic kyphosis has been reported to be linked to negative health effects, such as reduced lung and physical function, poor balance, falls, back pain, an increased risk of spinal fractures, a reduced quality of life, and earlier mortality [3]. With these adverse health effects, there is a need for effective preventative and therapeutic interventions targeting this condition.

Physiotherapists frequently encounter age-related hyperkyphosis in their practice [7,8,9]. This condition exhibits increased prevalence with advancing age, and studies estimate that 20% to 40% of older adults experience hyperkyphosis [2,3,10]. Furthermore, the acceptance of a prevalence rate ranging from 20% to 40% among elderly patients is widely acknowledged [11,12]. This prevalence increases with age, affecting up to 75.2% of older adults [9]. Given this high prevalence, physiotherapists’ knowledge of age-related hyperkyphosis plays a crucial role in preventing, treating, and caring for patients with hyperkyphosis, promoting the quality of life of the elderly, aiding them in maintaining their daily activities, and reducing health-related issues [13]. Treatment objectives aim to reduce the thoracic kyphosis angle, prevent complications, increase spinal mobility, and enhance quality of life. Therefore, a good understanding of this condition, along with proper attitude and confidence in its management, is crucial for physiotherapists. This expertise enables them to handle complex spinal deformities effectively and implement prevention programs for this population.

Despite the growing knowledge of age-related hyperkyphosis [14,15,16], physiotherapists have only recently started to pay attention to it [17]. However, previously physiotherapists have also shown a lack of significant interest in addressing the issue of thoracic hyperkyphosis, particularly in terms of their knowledge and attitudes towards the condition [7,17]. A study conducted in Australia by Perriman et al. (2012) surveyed the management of thoracic hyperkyphosis by Australian physiotherapists. The authors reported that physiotherapists needed to gain more knowledge regarding the management of hyperkyphosis [7].

In Thailand, the current knowledge of age-related hyperkyphosis, including its definition, prevalence, clinical assessment, gold-standard diagnosis, and appropriate treatment of hyperkyphosis in the elderly population among physiotherapists, is currently unknown. Therefore, the aims of this study were (1) to investigate the level of knowledge, attitudes, and confidence of age-related hyperkyphosis among Thai physiotherapists and (2) to compare them between experienced and inexperienced physiotherapists.

## 2. Materials and Methods

### 2.1. Research Design and Participants

This study was a cross-sectional, online survey, conducted between March 2023 and August 2023. Eligible respondents in this study were licensed physiotherapists. The inclusion criteria of the respondents were (1) being a licensed physiotherapist and (2) full comprehension of the Thai language. The exclusion criterion was an inability to complete the questionnaire. Participants who were recruited were categorized into two groups: experienced and inexperienced physiotherapists. In the context of this research, physiotherapists were considered experienced if they had prior experience in managing age-related hyperkyphosis. Those who had never had experience in this particular field were identified as inexperienced.

The sample size for this study was calculated using an online sample size calculator (available at https://www.calculator.net/sample-size-calculator.html, accessed on 18 October 2022), with the following assumptions: a 5% margin of error and a 50% response distribution. The total number of licensed physiotherapists in Thailand is 13,204 (data from Thai Physiotherapy Council on 18 October 2022). The sample size calculations indicated that a minimum of 374 participants were required for this study.

### 2.2. Measures and Instruments

#### 2.2.1. Questionnaire Development and Pre-Testing

The questionnaire used for data collection was adapted from a previous study by Perriman et al. (2012) [7]. The content validity of the current questionnaire was evaluated by five physiotherapists specializing in age-related hyperkyphosis. The Item–Content Validity Index (I-CVI) calculations for the 25 items that were preserved following the initial assessment by the panelists exhibited values ranging from 0.6 to 1.00, thereby indicating that these items were deemed to be clear, comprehensible, and relevant to the questionnaire. If the index of the IOC is between 0.5 and 1.00, it suggests that the item is acceptable [18]. These experts each contributed to the final list by providing feedback on the survey’s structure, wording, question sequence, and content accuracy. A pilot study was conducted with 30 participants to assess the relevance and comprehensiveness of the questions, leading to revisions in the questionnaire.

#### 2.2.2. Questionnaire

The self-administered questionnaire comprised five sections. Section A (5 items) collected information regarding the demographic data of the respondents (e.g., age, gender, education level). Section B (8 items) collected information about professional status and work experience (specialty, years of experience, and number of patients with hyperkyphosis seen per week) related to the work experience of physiotherapists. Section C (10 items) investigated the knowledge of age-related hyperkyphosis (definition, causes, timing, prevalence, gold-standard diagnosis, prognosis, treatment) and included five single-choice questions and five multiple-choice questions. Section D (2 items) collected data on attitudes towards age-related hyperkyphosis (using a 5-point Likert scale from strongly disagree to strongly agree). Finally, section E (1 item) evaluated the self-rated confidence level in treating age-related hyperkyphosis (11-point rating scale, 0 = not confident and 10 = very confident) (Supplementary Materials).

#### 2.2.3. Data Collection Procedure

The questionnaire was distributed to licensed physiotherapists through an online survey system on the Google Form platform and was accessible from March 2023 for 6 months. This study was promoted widely throughout the 13 Thai health service regions, the Thai Physical Therapy Council, and the Physical Therapy Association of Thailand. It was also promoted through Facebook and was shared with the link through targeted posts on physiotherapy groups and pages. These posts included a brief explanation of the purpose of the survey, contact information for the research team, a detailed informed consent letter, and a link to the survey. Additionally, the posts encouraged participants to share the survey with their networks to increase reach and engagement.

### 2.3. Data Analysis

Statistical analysis was performed using SPSS (version 23-IBM Corporation, Armonk, NY, USA). Descriptive statistics, including frequency and percentage of responses from participants, were calculated. A chi-square test was conducted to compare the differences in the level of knowledge (questions 1–5), and an independent *t*-test was conducted to assess attitudes towards age-related hyperkyphosis and confidence in treating it. A *p*-value of <0.05 was considered statistically significant.

## 3. Results

### 3.1. Participants’ Characteristics

A total of 314 physiotherapists completed the questionnaire. Close to 76% were female, and 39.49% of the participants were aged between 21 and 30. Most (85.35%) were practicing physiotherapists. More than half (65.92%) had a bachelor’s degree, and 54.46% had an orthopedic specialty. Nearly half of the respondents (41.47%) worked in a public hospital. Geographically, 47.45% practiced in Bangkok and its surroundings. About one-third (33.43%) had been practicing for between 5 and 10 years. Close to 68% of the participants had never treated or had experience with age-related hyperkyphosis, and 51% of the respondents treated older adults with hyperkyphosis once a week. Among participants with experience treating this condition, most reported treating less than one patient per week (53%). Almost all respondents (91.72%) had not undergone post-qualification training in this field (Table 1).

### 3.2. Knowledge

More than half of the respondents (68.79%) correctly answered the question regarding the definition of thoracic hyperkyphosis. Most were knowledgeable (90%) regarding the causes of age-related hyperkyphosis. However, only 14.97% of the respondents correctly answered the question on the age of onset and the prevalence (20.38%) of age-related hyperkyphosis. Over half of the respondents (64.65%) correctly answered the gold-standard diagnosis question regarding age-related hyperkyphosis. Nearly half of the respondents (43.31%) reported using visual examination to diagnose thoracic hyperkyphosis. The majority of the respondents (94.27%) knew that impaired pulmonary function could be affected by age-related hyperkyphosis, and 88.85% of the respondents reported that the main aim of treatment was to increase spinal mobility and flexibility. Nearly half of the respondents (47.45%) used corrective postural exercises to treat age-related hyperkyphosis. Among the respondents, 56.05% mentioned using the knowledge gained from undergraduate education (Table 2).

The overall mean score of correct answers was 2.59 ± 1.14 (out of 5). There was no statistically significant difference in the number of correct answers between experienced and inexperienced physiotherapists (2.65 ± 1.10 and 2.57 ± 1.15, *p* = 0.540, respectively).

Table 3 provides a comparison of the knowledge levels regarding age-related hyperkyphosis for each question from 1 to 5 between experienced and inexperienced physiotherapists. There were no statistically significant differences between experienced and inexperienced physiotherapists regarding the knowledge of the definition, age of onset, prevalence, and the gold-standard diagnosis of age-related hyperkyphosis (*p* > 0.05). Only the question relating to the age-related causes of hyperkyphosis showed a statistically significant difference between the two groups. Experienced physiotherapists had a higher number of correct answers than inexperienced physiotherapists (experienced physiotherapists = 97% vs. inexperienced physiotherapists = 87.38%; *p* = 0.007).

### 3.3. Attitudes

**Figure 1:**
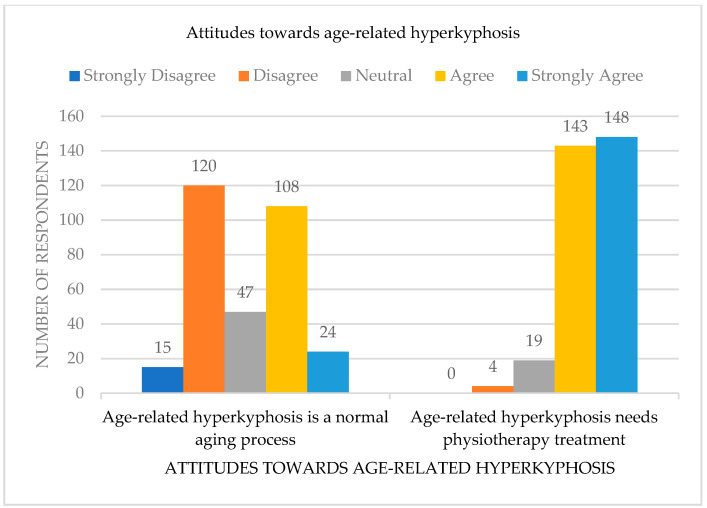
Attitudes towards age-related hyperkyphosis (*n* = 314). Number of survey respondents agreeing, being neutral, and disagreeing with statement about age-related hyperkyphosis.

There was almost an equal number of respondents who reported negative (43%, disagree = 38.22%, strongly disagree = 4.78%) and positive (42%, agree = 34.39%, strongly agree = 7.64%) attitudes towards the statement that age-related hyperkyphosis is a normal part of aging. Almost all respondents (92.67%) reported a positive attitude (agree = 45.54% and strongly agree = 47.13%) towards the statement that elderly individuals with hyperkyphosis need to receive physiotherapy treatment (Figure 1). There were no significant differences between experienced and inexperienced physiotherapists regarding attitudes towards the statement that “age-related hyperkyphosis is a normal part of aging”. There were significant differences between experienced and inexperienced physiotherapists concerning their attitudes towards the statement that elderly individuals with hyperkyphosis need to receive physiotherapy treatment (Table 4).

### 3.4. Self-Rated Confidence

The average level of self-rated confidence in treating age-related hyperkyphosis, measured on an 11-point Likert scale, where 0 indicates not confident and 10 indicates very confident, was moderate at 5. In addition, the median self-rated confidence in assessing and treating age-related hyperkyphosis was five (25th, 75th quartiles: 3–6.25). The highest percentage of the respondents (22.61%) reported having a moderate level of self-rated confidence in treating age-related hyperkyphosis. In contrast, few respondents reported their confidence in providing treatment for this condition as being very low and scored one. Only 0.96% of the respondents reported having a self-rated confidence rating at level 10. As expected, experienced physiotherapists reported having a higher confidence in assessing and treating age-related hyperkyphosis than inexperienced physiotherapists (5.81 ± 2.02 vs. 4.11 ± 2.22; *p* < 0.001; 95% confidence interval is 1.19–2.22) (Table 5).

## 4. Discussion

### 4.1. Key Findings

To the best of our knowledge, this study is the first to examine the extent of knowledge, attitudes, and confidence regarding age-related hyperkyphosis among Thai physiotherapists. The main findings of this study were that physiotherapists had a “good” understanding of the causes of age-related hyperkyphosis but relatively moderate knowledge of the definition and diagnosis methods and less knowledge of the age of onset and prevalence of hyperkyphosis. Nearly half of them reported the use of visual examination to diagnose this condition. Most physiotherapists reported that age-related hyperkyphosis affected pulmonary function and identified that the main objective of treating this condition was to improve spinal mobility and flexibility. Almost half of the respondents chose corrective posture exercises as the treatment modality. Over half of the respondents utilized their undergraduate education in orthopedics to address age-related hyperkyphosis. Regarding the attitudes, an almost equal proportion of the participants expressed negative and positive perspectives towards the idea that age-related hyperkyphosis is part of the aging process. Almost all respondents reported having a concurring attitude towards the statement that elderly individuals with hyperkyphosis required physiotherapy treatment. As expected, experienced physiotherapists had a higher number of correct answers than inexperienced physiotherapists. Both experienced and inexperienced physiotherapists showed differences in their attitudes towards the suggestion that elderly individuals with hyperkyphosis needed physiotherapy treatment. In addition, nearly one-quarter of the physiotherapists had a moderate level of self-rated confidence in treating age-related hyperkyphosis. Furthermore, experienced physiotherapists had a higher level of confidence in managing this condition than inexperienced physiotherapists, as expected.

### 4.2. Epidemiology

The current study found that 68.79% of the respondents answered the question about the definition correctly, indicating a moderate understanding of its definition. Although a kyphosis of 40 degrees or more is commonly accepted as the definition of thoracic hyperkyphosis, there is still no standard definition of thoracic hyperkyphosis due to a variety of different kyphosis measurement methods found in practice and in the literature [14]. This may be why the respondents answered this question incorrectly.

Most respondents answered the question about the causes correctly, demonstrating a good understanding of this condition. A recent study conducted by Woods et al. found that older men with thoracic hyperkyphosis were associated with low body mass index, bone mass density, family history of thoracic hyperkyphosis, and prevalent vertebral fractures, with degenerative disk disease influencing kyphosis progression [19]. Understanding the causes of age-related hyperkyphosis allows physiotherapists to delay its progression and take preventive measures.

Only 14.97% of the respondents correctly answered the question about the age of onset, indicating that most respondents had limited knowledge. In age-related hyperkyphosis, older adults experience a significant increase in thoracic curvature in both males and females after the age of 40 [1,20], and this tends to progress with age [10]. Ailon et al. [10] stated that the mean angle of kyphosis increases from 43° in females aged 55–60 to 52° in those aged 76–80. Thus, recognizing individuals at high risk of thoracic hyperkyphosis is crucial for prevention.

Only 20% of the respondents answered the question about prevalence correctly, indicating a lack of knowledge about this aspect of hyperkyphosis. Unfortunately, despite a thorough search, no other study has reported on the level of knowledge about prevalence. It was difficult to compare the results of the current study with previously published results because of the lack of comparable data. The exact prevalence has not yet been established. Studies conducted by various researchers, such as Katzman et al. [3], Li et al. [8], and Bimali et al. [9], have documented the prevalence of thoracic hyperkyphosis in the elderly population to be between 20% and 75% across different nations. It is possible that the prevalence of this condition varies among countries.

Over half of the respondents answered the question about age-related hyperkyphosis using gold-standard methods correctly, indicating that physiotherapists had a good understanding of this topic. This typically involves utilizing the Cobb method from radiographic images, where the angle of the thoracic spine is greater than 40–50 degrees in the sagittal plane. Unfortunately, the physiotherapy clinic’s staff lacks direct access to X-ray imaging as they are not authorized to order them themselves.

Regarding non-invasive kyphosis measurement, nearly half of the respondents reported using visual examination. This preference for visual examinations may be convenient and does not require any equipment. This finding is consistent with that of Perriman [7], who reported that physiotherapists assess age-related hyperkyphosis primarily through visual examination. Relying solely on visual examination may lead to subjective assessments and difficulty in monitoring the progress of the disease because visual methods are not accurate or evidence-based. Therefore, visual examination alone may not provide an accurate diagnosis. Therefore, additional assessment methods are necessary to ensure an accurate diagnosis and easier monitoring of patients with hyperkyphosis. Furthermore, Isherwood et al. [21] suggested that physiotherapists should conduct a thorough postural assessment, incorporating both subjective and objective evaluations.

Almost all respondents reported that age-related hyperkyphosis affected the respiratory system. This indicates that physiotherapists are concerned about serious complications and side effects. The respondents also reported other complications, including impaired balance, back pain, loss of self-image, and an increased risk of vertebral fractures. A similar study by Sran and Khan [22] expressed concerns regarding the increased incidence of vertebral fractures as a complication of osteoporosis. This raises doubts about the effectiveness of manual therapy as a treatment for physiotherapy. Knowledge of these side effects and/or complications may help inform the development and efficacy of hyperkyphosis management and help prevent health-related issues [23].

Most of the respondents identified the primary goal of treating age-related hyperkyphosis as improved spinal mobility and flexibility, followed by postural re-education with the aim of increasing muscular retraction strength. These findings, as supported by Bettany-Saltikov et al. [24], suggest that exercises focusing on strengthening the spinal muscles and improving spinal mobility may help decrease kyphosis angle. When combined with postural training, these exercises may help older adults maintain an upright posture. Treatment goals need to be clearly defined to effectively address and reduce the kyphosis angle, improve the strength of the back muscles, and slow down the progression and negative consequences of hyperkyphosis [10].

Regarding treatment methods, nearly half of the respondents chose corrective posture exercises for treatment in this survey. Approximately one-third of the respondents used postural re-education as a treatment. The results of this study agree with those of Perriman et al. [7], who demonstrated that most of the participants primarily used postural re-education for treatment. Schoenfeld et al. [25] conducted a survey on the most current methods for treating post-traumatic kyphosis in a multinational group of spinal trauma surgeons. They reported that surgeons most often suggest pain medication and physical therapy for non-operative treatment. A recent systematic review and meta-analysis by Ponzano et al. (2021) suggested that exercise programs focusing on hyperkyphosis, such as strengthening the back extensor muscles, could potentially enhance the kyphosis angle and muscle strength in older adults with hyperkyphosis [15].

Over half the respondents used their undergraduate knowledge to manage age-related hyperkyphosis. This indicates a potential gap in the continuous development of professional knowledge and may indicate outdated knowledge or practices in the field. This aligns with the results of Perriman et al. [7], indicating that physiotherapists often use their undergraduate knowledge in practice. Given that the complexity of this condition requires expertise beyond the scope of typical undergraduate education, it may not be sufficient for effective management. Thus, postgraduate courses for the treatment of spinal deformities are warranted.

### 4.3. Attitudes

An equivalent proportion of respondents had both negative and positive views on whether age-related hyperkyphosis is a normal aging process. One reason for this may be that age-related hyperkyphosis is believed to be part of the normal aging process or geriatric syndrome [26,27].

Most respondents supported the idea that older individuals with hyperkyphosis require physiotherapy. The results align with those of Korakakis et al. [28], who found that most physiotherapists believed that maintaining an upright lordotic lumbar spinal posture, whether sitting or standing, was ideal. This highlights the importance of physiotherapy in promoting proper posture. These findings were supported by Ponzano et al. [15], Jenkins et al. [16], and Duangkaew et al. [29], who raised awareness of this deformity and focused on potentially effective treatments for age-related hyperkyphosis [15,16,29]. Furthermore, Desdiani and Sari [30] suggested that physiotherapists consider severe age-related hyperkyphosis as an abnormal posture that requires early detection and corrective interventions to prevent complications.

### 4.4. Confidence

More than half of the respondents had a moderate-to-high level of confidence in treating age-related hyperkyphosis, while 41.09% reported having low confidence in treating patients with hyperkyphosis, with levels ranging from 1 to 4. This finding suggests that the respondents lacked confidence in treating this condition. This may be because most surveyed physiotherapists lacked experience in assessing and treating age-related hyperkyphosis. However, research on physiotherapists’ confidence in managing age-related hyperkyphosis is limited.

### 4.5. Comparing the Level of Knowledge, Attitudes, and Confidence between Experienced and Inexperienced Physiotherapists

The level of knowledge of the definition, age of onset, prevalence, and gold-standard of diagnosis of age-related hyperkyphosis between experienced and inexperienced physiotherapists was not significantly different except for knowing the cause. This indicated that both experienced and inexperienced physiotherapists had insufficient knowledge and understanding of age-related hyperkyphosis. To the authors’ knowledge, no previous studies have compared the level of knowledge regarding age-related hyperkyphosis. Therefore, comparing this study’s findings with other studies was not possible. A similar study on idiopathic scoliosis by Du Toit et al. conducted an online survey to compare the level of knowledge on idiopathic scoliosis between physiotherapists who were registered as orthopedic manipulative physiotherapist and those who were not registered but were interested in orthopedic and musculoskeletal physiotherapy. The authors reported that the level of knowledge on conservative treatment was not significantly different between the groups, and the overall correct responses from both groups were low [31].

The attitudes towards age-related hyperkyphosis as a normal aging process did not differ between experienced physiotherapists and inexperienced physiotherapists. Experienced physiotherapists had a more positive attitude towards the importance of physiotherapy treatment for age-related hyperkyphosis than inexperienced physiotherapists. Moreover, as expected, experienced physiotherapists had higher levels of confidence in treating age-related hyperkyphosis compared to inexperienced physiotherapists. A possible explanation for this is that physiotherapists are only now beginning to recognize age-related hyperkyphosis as a significant health problem [32]. In addition, as a result, it can be anticipated that experienced physiotherapists who are familiar with hyperkyphosis patients will demonstrate greater efficacy in their approaches.

### 4.6. Implications

The current study reveals significant gaps in knowledge, particularly regarding the age of onset and prevalence of age-related hyperkyphosis. These gaps indicate a need for targeted education and training to enhance physiotherapists’ skills to manage this condition effectively. The findings also suggest that physiotherapists could refine their management of age-related hyperkyphosis by offering webinars or training courses to improve their understanding of effective intervention strategies and enhance patient outcomes. Therefore, physiotherapists must engage in continuous professional development through independent self-learning such as reading, research, attending workshops, and engaging in professional training to enhance their knowledge and skills consistently. Additionally, understanding age-related hyperkyphosis could be a beneficial addition to the undergraduate physiotherapy curriculum.

### 4.7. Limitations of This Study

This study has some limitations. Firstly, using an online questionnaire limited the depth of information required from respondents. Secondly, the findings of this study were based on self-reported information, raising the possibility of recall bias that could have affected the outcome. Thirdly, the findings may not apply to other countries with different education structures and healthcare systems. Fourthly, a self-selection bias may limit the generalizability to the entire Thai physiotherapist population. Fifth, the lack of adjustment for potentially confounding variables such as age and gender may affect the results. Finally, the absence of a website visit counter made it challenging to assess the survey invitation’s exposure level and collect data online. Therefore, response rates could not be computed. In future studies, it is recommended that qualitative research be conducted to gain a deeper understanding of the knowledge, attitudes, and levels of confidence of physiotherapists regarding age-related hyperkyphosis. In addition, future studies could focus on respondents with direct experience in treating age-related hyperkyphosis to provide a more targeted analysis. Furthermore, educational interventions could be implemented and assessed for their impact on confidence and attitudes.

## 5. Conclusions

In conclusion, Thai physiotherapists appear to lack an overall understanding of age-related hyperkyphosis and feel ambivalent about how the aging process might affect it. Nonetheless, most of them agreed on the need for treatment. Experienced physiotherapists had more confidence in treating this condition and a better attitude towards aging than inexperienced physiotherapists. The findings of this study support the importance of raising awareness of all aspects of age-related hyperkyphosis among Thai physiotherapists, likely leading to the better management of older adults with this condition. Finally, undergraduate curricula could be assessed to address these knowledge gaps to see how this condition is presented to students, and postgraduate training could be implemented.

## Figures and Tables

**Table 1:** Demographic of survey respondents (*n* = 314).

Characteristic	Survey Respondents (%)
**Gender**	
Male	68 (21.66)
Female	238 (75.79)
Non-binary	8 (2.55)
**Age range (y)**	
21–30	124 (39.49)
31–40	109 (34.71)
41–50	75 (23.89)
51–60	6 (1.91)
**Job position**	
Physiotherapist	268 (85.35)
Physiotherapy professor	34 (10.83)
Other	12 (3.82)
**Education level/degree**	
Bachelor’s degree	207 (65.92)
Master’s degree	77 (24.52)
PhD	20 (6.37)
Postdoctoral	5 (1.59)
Graduate diploma	5 (1.59)
**PT specialty (may select more than one) ^a^**	
Orthopedics	171 (54.46)
Neurology	101 (32.17)
Cardiopulmonary system	49 (15.61)
Pediatrics	27 (8.60)
Sport	27 (8.60)
Geriatrics	31 (9.87)
other	17 (5.14)
**Practice setting (may select more than one) ^a^**	
Public hospital	131 (41.72)
Private hospital	53 (16.88)
University hospital	24 (7.64)
University clinic	48 (15.29)
Outpatient private clinic	50 (15.92)
Sport club	13 (4.14)
Other	24 (7.64)
**Region of workplace**	
Bangkok and its vicinity	149 (47.45)
Northern	34 (10.83)
Central	47 (14.97)
Southern	31 (9.87)
Northeastern	19 (6.05)
Eastern	25 (7.96)
Western	9 (2.86)
**Years of clinical practice**	
0–5	94 (29.94)
5–10	105 (33.43)
10–15	37 (11.78)
15–20	56 (17.83)
>20	22 (7.00)
**Experience in treating age-related hyperkyphosis**	
Yes	100 (31.85)
No	214 (68.15)
**Frequency of treating age-related hyperkyphosis (n = 100)**	
Everyday	4 (4)
Once a week	51 (51)
Once a month	17 (17)
Once a year	28 (28)
**Number of hyperkyphotic patients/week** **(n = 100)**	
<1	53 (53)
1–4	41 (41)
5–10	5 (5)
>10	1(1)
**Additional training on the treatment of age-related hyperkyphosis**	
Yes	26 (8.28)
No	288 (91.72)

PhD: Philosophy Doctorate. ^a^ Totals for the survey data may not equal exactly 100% due to rounding to one digit to the right of the decimal point.

**Table 2:** Respondents’ knowledge about age-related hyperkyphosis (*n* = 314).

Knowledge Questions (Q1–Q5)	Number of Correct Answers	
	*n*	(%)
Q 1. Definition of thoracic hyperkyphosis	216	68.79
Q 2. Causes of age-related hyperkyphosis	284	90
Q 3. Age of onset of hyperkyphosis	47	14.97
Q 4. Prevalence of age-related hyperkyphosis	64	20.38
Q 5. Gold-standard diagnosis of age-related hyperkyphosis	203	64.65
Q 6. Do you have experience in using any of the following non-invasive methods to diagnose thoracic hyperkyphosis? (may select more than one)		
1.7 cm block	3	1
Cobb method	52	15.25
Electrogoniometer	3	1
Flexicurve ruler	29	9.24
Inclinometer	27	8.6
Occipital to wall distance	66	21.02
Photography	17	5.41
Plurimeter	0	0
Visual examination	136	43.31
Q 7. What is the negative impact of age-related hyperkyphosis? (may select more than one)		
Increased risk of fall	266	84.71
Activities of daily living limitation	262	83.44
Impaired pulmonary function	296	94.27
Back pain	250	79.62
Loss of their self-image	248	78.98
Impaired balance	271	86.31
Increase risk of vertebral fractures	182	57.96
Q 8. What are the objectives of treating age-related hyperkyphosis? (may select more than one)		
Increase spinal mobility and flexibility	279	88.85
Increase muscular retraction strength	238	75.80
Postural re-education and implementation in daily activities	261	83.12
Increase back muscle endurance	204	64.97
Reduce Cobb angle	156	49.68
Q 9. What treatment techniques of conservative treatment of thoracic hyperkyphosis are you familiar with? (may select more than one)		
Hydrotherapy	48	15.29
Postural taping	28	8.92
Frenkel’s training	8	2.55
Postural stretching	132	42.04
Spinal mobilization	73	23.25
Postural re-education	118	37.58
Back-strengthening exercises	143	45.54
Corrective posture exercises	149	47.45
Schroth best practice program	22	7.01
Alexander-based corrective techniques	1	0.32
International Schroth 3D scoliosis therapy (ISST)	8	2.55
Never treat age-related hyperkyphosis	143	45.54
Q 10. On what basis do you formulate your decision regarding the diagnosis and treatment in older adults with thoracic hyperkyphosis? (may select more than one)		
Undergraduate education	176	56.05
Directly conducted clinical research in this particular domain in this area	12	3.82
Professional training	45	14.33
Own reading of the literature	97	30.89
Never assess/treat age-related hyperkyphosis	127	40.45

**Table 3:** Comparison of knowledge about age-related hyperkyphosis between experienced physiotherapists and inexperienced physiotherapists (questions 1–5) (chi-square test) (*n* = 314).

Knowledge Questions	Number of Correct Answers		
	Experienced Physiotherapists (*n* = 100), *n* (%)	Inexperienced physiotherapists (*n* = 214), *n* (%)	*p*-Value
Q 1. Definition of thoracic hyperkyphosis	66 (66)	150 (70.90)	0.466
Q 2. Causes of age-related hyperkyphosis	97 (97)	187 (87.38)	0.007 *
Q 3. Age of onset of hyperkyphosis	18 (18)	29 (13.55)	0.303
Q 4. Prevalence of age-related hyperkyphosis	19 (19)	45 (21.03)	0.678
Q 5. Gold-standard diagnosis of age-related hyperkyphosis	65 (65)	138 (43.95)	0.929

* *p* < 0.05.

**Table 4:** Comparison of attitudes towards age-related hyperkyphosis between experienced physiotherapists and inexperienced physiotherapists (*n* = 314).

Attitudes	Experienced Physiotherapists *n* = 100	Inexperienced Physiotherapists *n* = 214	*p*-Value
Age-related hyperkyphosis is a normal aging process			
Median (25th, 75th quartiles)	4 (2–4)	3 (2–4)	NA
Mean ± SD	3.05 ± 1.16	2.95 ± 1.08	0.451
Age-related hyperkyphosis needs physiotherapy treatment			
Median (25th, 75th quartiles)	5 (4–5)	4 (4–5)	NA
Mean ± SD	4.55 ± 0.59	4.31 ± 0.68	0.002 *

SD: standard deviation; * *p* < 0.05.

**Table 5:** Level of confidence in treating age-related hyperkyphosis.

Level of Confidence	*n* = 314	%
0	0	0
1	48	15.29
2	15	4.78
3	42	13.38
4	24	7.64
5	71	22.61
6	36	11.46
7	47	14.97
8	22	7.01
9	6	1.91
10	3	0.96

## Data Availability

The data that support the findings of this study are available from the corresponding author (R.D.) upon reasonable request. These data, due to confidentiality and ethical considerations, are not publicly available.

## Supplementary Materials

The following supporting information can be downloaded at: https://www.mdpi.com/article/10.3390/healthcare12191998/s1, Table S1: Questionnaire about knowledge and attitude regarding age-related hyperkyphosis among Thai physiotherapists.
