# Supplementary material for: Survey of Knowledge, Attitudes, and Levels of Confidence Regarding Age-Related Hyperkyphosis and Its Management among Thai Physiotherapists
Source: Healthcare (Basel). 2024 Oct 7;12(19):1998. doi: 10.3390/healthcare12191998 (PMC11477387; doi:10.3390/healthcare12191998)
Supplement: Supplementary file 1 [file healthcare-12-01998-s001.zip › healthcare-3170588-supplementary.pdf]

Supplementary material for Duangkaew et al. Survey of Knowledge, Attitudes, and Levels of Confidence Regarding Age-Related Hyperkyphosis and Its Management among Thai physiotherapists

The questionnaire of knowledge and attitude regarding age-related hyperkyphosis among Thai physiotherapists

Section A: Demographic data

I would like to ask you questions related to your personal.

1.Sex

☐ Male

☐ Female

☐ Non-binary

2. Age (year)

☐ 21 - 30

☐ 31 - 40

☐ 41 - 50

☐ 51 - 60

☐ Over 60

3. Job position

☐ Physiotherapist

☐ Physiotherapy professor

☐ Other

4. What is the highest level of education that you have achieved?

☐ Bachelor's degree

☐ Master's degree

☐ PhD

☐ Postdoctoral

☐ Graduate diploma

5.Additional training on the treatment of age-related hyperkyphosis

☐ Yes (please specify)…………………………………………

☐ No

Section B: Professional status and work experience

1. PT specialty (may select more than one)

    ☐ Orthopedics

    ☐ Neurology

    ☐ Cardiopulmonary system

    ☐ Pediatrics

    ☐ Sport

☐ Geriatrics

☐ Other……………………………………………………………………………………….

2. Practice setting (may select more than one)

☐ Public hospital

☐ Private hospital

☐ University hospital

☐ University clinic

☐ Outpatient private clinic

☐ Sport club

☐ Other……………………………………………………………………………………….

3. Region of workplace

☐ Bangkok and its vicinity

☐ Northern

☐ Central

☐ Southern

☐ Northeastern

☐ Eastern

☐ Western

4. Years of clinical practice (year)

☐ 0-5

☐ 5-10

☐ 10-15

☐ 15-20

☐ >20

5. Experience in treating age-related hyperkyphosis

☐ Yes ……………………………...

☐ No

6. How often do you treat age-related hyperkyphosis?

☐ Everyday

☐ Once a week

☐ Once a month

☐ Once a year

☐ Never treat hyperkyphosis

7. If your answer "NO" in Item No.6, please select the answer below

☐ I had never treated patient (not older adults) with thoracic hyperkyphosis

☐ I had never treated older adults with thoracic hyperkyphosis

☐ Other……………..……………..……………..…

8. Number of hyperkyphotic patients/week

☐ 0

☐ < 1

☐ 1- 4

☐ 5-10

☐ >10

Section C: The knowledge of age-related hyperkyphosis (definition, causes, timing, prevalence, gold-standard diagnosis, prognosis, treatment) and included five single-choice questions and five multiple-choice questions

Please select ONLY ONE answer for the question 1 to question 5

1. What is age-related hyperkyphosis?

☐ The thoracic spine has an abnormal curvature, leading to compensatory changes in other parts of the body such as rounded shoulder, forward head posture.

☐ The thoracic spine has an abnormal curvature combined with the displacement of the head anteriorly, resulting in a cranio-vertebral angle of less than 53 degrees.

☐ An excessive anterior curvature of the thoracic spine greater than 40° - 50° using the Cobb Method.

☐ An excessive anterior curvature of the lumbar spine greater than 40° - 50° using the Cobb Method.

☐ I do not know.

2. What causes age-related hyperkyphosis?

☐ Unknow causes

☐ Abnormalities in spine development during childhood can lead to symptoms that manifest in older adults.

☐ Age-related hyperkyphosis is a spinal deformity caused by Scheuermann's disease.

☐ Age-related hyperkyphosis has a multifactorial etiology, such as degenerative disc and vertebral disease, osteoporosis, back extensor weakness, prolonged abnormal posture.

☐ I do not know

3. When does age-related hyperkyphosis develop?

☐ 30 – 39 years old

☐ 40 – 49 years old

☐ 50 – 59 years old

☐ 60 – 69 years old

☐ I do not know

4. How prevalent is age-related hyperkyphosis?

☐ Approximately 5-10

☐ Approximately 10-30

☐ Approximately 20-40

☐ Approximately 30-50

☐ I do not know

5. How is the gold standard diagnosis of age-related hyperkyphosis?

☐ Assess kyphosis angle by visually examining the posture of patients. Look for signs such as a forward head, rounded shoulders, increased thoracic kyphosis, hip flexion, and knee flexion.

☐ A kyphosis angle greater than 40°-50°, measured using the Cobb method on a standing lateral spinal x-ray.

☐ The Cobb method is used to measure the angle of kyphosis, often along with evaluating vertebral fractures.

☐ A kyphosis angle greater than 10° combined with the rotation of spinal vertebrae, measured using the Cobb method on a standing lateral spinal x-ray.

☐ I don't know

6. Do you have experience in using any non-invasive method to diagnose thoracic hyperkyphosis? (may select more than one)

☐ Plurimeter

☐ Inclinometer

☐ Photography

☐ Flexicurve ruler

☐ Electrogoniometer

☐ 1.7 cm block

☐ Visual examination

☐ Occipital to wall distance

☐ Cobb method from radiographic

☐ Other……………………………………………………………………………………..

☐ No experience in assessing thoracic kyphosis

7. What is the negative impact of age-related hyperkyphosis? (may select more than one)

☐ Increase risk of fall

☐ Activities of daily living limitation

☐ Impaired pulmonary function

☐ Back pain

☐ Loss of their self-image

☐ Impaired balance

☐ Increase risk of vertebral fractures

8. What are the objectives of treating age-related hyperkyphosis? (may select more than one)

☐ Reduce Cobb angle

☐ Increase back muscle endurance

☐ Postural re-education and implemented in daily activities

☐ Increase muscular retraction strength

☐ Increase spinal mobility and flexibility

9. What treatment technique of conservative treatment of thoracic hyperkyphosis are you familiar with? (may select more than one)

☐ Hydrotherapy

☐ Postural taping

☐ Frenkel's training

☐ Postural stretching

☐ Spinal mobilization

☐ Postural re-education

☐ Back strengthening exercises

☐ Corrective posture exercises

☐ Schroth Best Practice Program

☐ Alexander Based Corrective techniques

☐ International Schroth 3-dimensional scoliosis therapy (ISST)

☐ Other……………………………………………………………………………………………..

☐ Never treat age-related hyperkyphosis

10. On what basis do you formulate your decision regarding the diagnosis and treating older adults with thoracic hyperkyphosis? (may select more than one)

☐ Undergraduate education

☐ Directly conducted clinical research in this particular domain in this area

☐ Professional training

☐ Own reading of the literature

☐ Other……………………………………………………………………………………………..

☐ Never assess/or treating age-related hyperkyphosis

Section D: Attitudes towards age-related hyperkyphosis

I would like to ask questions about your views on age-related hyperkyphosis. I am referring to your feelings about the questions I will ask.

1. Attitudes towards age-related hyperkyphosis is a normal aging process

☐ Strongly agree

☐ Agree

☐ Neutral

☐ Disagree

☐ Strongly disagree

2. Attitudes towards age-related hyperkyphosis needs physiotherapy treatment

☐ Strongly agree

☐ Agree

☐ Neutral

☐ Disagree

☐ Strongly disagree

Section E: The self-rated confidence level in treating age-related hyperkyphosis

How would you rate your confidence in treating age-related hyperkyhposis?

	1	2	3	4	5	6	7	8	9	10	
Not confident	❍	❍	❍	❍	❍	❍	❍	❍	❍	❍	Very confident
